# Emotional intelligence: Its place in infection prevention and control

**DOI:** 10.1177/17571774231159573

**Published:** 2023-03-08

**Authors:** Mark Cole

**Affiliations:** 5292 University of Manchester, Manchester, UK

**Keywords:** competence, emotional intelligence, infection prevention and control

## Abstract

**Background:**

The Infection Prevention Societies Competency Framework is a detailed tool that recognises the multi factorial work of Infection Prevention and Control Teams. This work often takes place in complex, chaotic and busy environments where non-compliance with policies, procedures and guidelines is endemic. As reductions in Healthcare Associated Infection became a health service priority the tone of Infection Prevention and Control (IPC) became increasingly uncompromising and punitive. This can create conflict between IPC professionals and clinicians who may take a different view as to the reasons for sub optimum practice. If unresolved, this can create a tension that has a negative impact on working relationships and ultimately patient outcomes.

**Concepts and Context:**

Emotional Intelligence, that ability to recognise, understand and manage our own emotions and recognise, understand and influence the emotions of others, is not something, hitherto, that has been headlined as an attribute for individuals working in IPC. Individuals with higher level of Emotional Intelligence show a greater capacity for learning, deal with pressure more effectively, communicate in interesting and assertive ways and recognise the strengths and weaknesses of others. Overall, the trend is that they are more productive and satisfied in the workplace.

**Conclusion:**

Emotional Intelligence should be a much sought after trait in IPC as this will better equip a post holder to deliver challenging IPC programmes. When appointing to an IPC team, the candidates Emotional Intelligence should be considered and then developed through a process of education and reflection.

## Introduction

The NHS reforms of the 1990s, the media representations of MRSA, public concern and increased patient expectations combined to raise the profile of Infection Prevention and Control (IPC) and made reducing the burden of Healthcare Associated Infection a health service priority (Royal College of Nursing and Infection Prevention Society, 2016). There followed an unprecedented investment in IPC nurses, ostensibly because they have a pivotal part to play in the delivery of an organisations IPC agenda. As a Clinical Nurse Specialist, the role is characterised by clinical acumen, expert knowledge, competence and autonomous practice and substantive areas of responsibility include the management and care of complex and vulnerable populations, education and support of inter-professional staff, and facilitation of change (Kilpatrick et al., 2014). The role of the Infection Control Professional (ICP) has become significantly more standardised and clarified since its inception and opinion leaders have made some notable achievements including the creation of competency standards for use at a national level. First developed in 2001, the competency framework of 2011 is the third and most detailed to date and has been called a milestone in the evolution of IPC in the United Kingdom (Burnett et al., 2011). Interestingly the framework does not make a reference to the Emotional Intelligence of an ICP. This short paper will consider why this should receive greater attention.

## Competency framework

The Competency Framework is built around four domains that include, Clinical Practice, Quality Improvement and Research, Education and Leadership and Management. It takes the practitioner through three levels of competence where they can be assessed as assisted, supervised or independent. It is a layered, diverse, tool that reflects the multifactorial nature of the ICP. While it does include the ‘softer’ aspects of performance such as the ability to communicate, build relationships, form partnerships, support, encourage and influence others, contemporary IPC has established something of a culture of a zero tolerant, deductive, top down approach to practice improvement. A position where best practice is understood and care needs to be delivered to a pre-planned end point that achieves the known outcome or target. Perhaps it is then not surprising that the framework is steeped in language that Storr et al. (2013) calls the traditional ‘weapons’ in the infection prevention ‘armoury’; for example, biological sciences, epidemiology, surveillance, education, policies, audit and legal frameworks.

## Emotional intelligence

Within the Competency Framework, there is no explicit reference to Emotional Intelligence (EI) as a competence, performance indicator or knowledge, understanding and skills that an IPC should hold and/or develop. To put a complex theory in simple terms, EI is the ability to perceive, evaluate and manage emotions in oneself, other people and groups. It is that ability to know what to say and when, why, how, or how not, to engage with others. High levels of EI have been found to be related to workplace performance in highly demanding work environments and as a consequence successful organisations recognise its importance, recruit those who possess it and promote it on a daily basis to facilitate career growth (Gilar-Corbi et al., 2019). Some characteristics of individuals with high and low EL can be seen in Table 1. The central tenets of EI can be seen in Table 2.

**Table 1.** Characteristics of Emotional Intelligence.

People with low EQ	People with high EQ
Often feels misunderstood	Understand the links between their emotions and how they behave
Get upset easily	Remain calm and composed during stressful situations
Become overwhelmed by emotions	Are able to influence others toward a common goal
Have problems being assertive	Handle difficult people with tact and diplomacy

(Harvard, 2019).

**Table 2.** Central Tenets of Emotional Intelligence.

Self-awareness
The ability to recognise and understand your moods ad emotions and how they affect others
Self-regulation
The ability to control impulses and moods and to think before acting
Internal motivation
Being driven to pursue goals for personal reasons rather than for some kind of reward
Empathy
The ability to recognise and understand others’ motivations and put yourself in their shoes
Social skills
The ability to manage relationships and build networks

Goleman (2020).

## Conflict and the IPC

To work at expert level specialist nurses need to harness the competencies of EI. The reason why EI resonates with IPC is that ICPs often discharge their responsibilities in environments that are complex and chaotic. Environments that are frequently associated with high workloads, poor staffing levels, a lack of time, language and cultural barriers, anxiety, pain and discomfort (Burnett, 2018). Although there may be an increasing acceptance that IPC is a collective responsibility where everyone is pulling in the same direction, In her study, Henderson et al. (2021) found an inconsistency between how IPCs and clinical staff viewed missed care and by association non-compliance in these stress laden environments. While clinicians would often cite antecedents around staffing, workload and patient acuity, IPCs would focus more on organisational and management factors. This divergence, if not addressed in a therapeutic manner, risks taking clinical practice into an area of conflict and tension. Brewster et al. (2016) argues that when IPC teams respond to the tension of missed care in a heavy-handed or confrontational way they can be resented and seen as a threatening presence on wards or units. According to Basogul and Ozgur (2016), these types of conflicts, whether they are functional or not, are essentially emotional because they arise from individuals’ or groups’ perception of threats to their agendas. If not managed, this can result in unhealthy working conditions and power games, which can then lead to patient dissatisfaction and a reduction in the quality of care.

It is not only their relationship with colleagues where ICPs need to draw of the tenets of EI. In her study, Harris et al. (2020) describe how nurses face challenges when providing sensitive information about multi-resistant organisms and related hospital policies, to patients who are found to be colonised. As an expert resource, the IPC is more likely to be called upon when these challenges are at their most acute. Their support may also be required when more junior colleagues become concerned for their own safety (Harris et al., 2020). In addition, ICPs may have an internal conflict when they advocate policies they do not necessarily agree with but follow under the banner of collective responsibility. Some decisions have an ethical component. For example, isolation is a common component of contact precautions to help interrupt the chain of infection. However, this rarely benefits the individual who is in isolation; indeed a meta-analysis into quality of life found isolation precautions may be associated with higher risks of anxiety, depression and several other psychological measures (Sharma et al., 2020). The tension between evidence-based practice and reasoned rationing of care possibly reached a new peak during the current COVID-19 pandemic where well established policies, procedures and guidelines become more ‘elastic’ overnight in the face of the impending crises. The scenarios above can stressful and exhausting, and lead to burnout. Enhancing EI skills would allow the ICP to better manage the emotional demands of their role, their behaviour and those of colleagues and patients.

## Conflict management strategies

Basogul and Ozgur (2016) go on to classify five different strategies that individuals use to handle interpersonal conflict in organisations (Table 3). In their study, the authors found that nurses would often choose an avoiding strategy. Typically, this means that they would attempt to distance themselves from conflict. An ICP, as a specialist, may not be typical of nurses as a whole and could select a different approach. Indeed as IPC, is often perceived as a highly regulated, zero tolerant and punitive discipline (Brewster et al., 2016) that holds the ‘moral authority’ of best practice, it is not inconceivable that a more dominating approach is adopted. While, to a point, this is understandable, avoiding and dominating strategies are sub optimum when managing conflict and are often associated with low levels of EI. By contrast, an integrating strategy, which is less common, is seen as a more effective way of managing conflict and is associated with a higher level of EI (Al-Hamdan et al., 2019).

**Table 3.** Conflict management strategies.

Integrating (high concern for self and others): This style involves openness, exchange of information and examination of differences to reach an effective solution acceptable to both parties. It is associated with problem solving, which may lead to creative solutions.
Obliging (low concern for self and high concern for others): This style is associated with attempting to play down differences and emphasising commonalities to satisfy the concerns of the other party.
Dominating (high concern for self and low concern for others): This style is identified with a win-lose orientation or with forcing behaviour to win someone’s or group’s position.
Compromising (intermediate concern for self and others): This style involves give-and-take, whereby both parties give up something to make a mutually acceptable decision.
Avoiding (low concern for self and others): This style is associated with withdrawal, buck-passing or sidestepping situations.

## Developing emotional intelligence

As scholars in IPC can attest, providing definitive evidence of the effectiveness of an intervention can be problematic because of limitations in research methodologies. However, in their comprehensive review of the literature, Gilar-Corbi et al. (2019) report that the indicators suggest that individuals with higher level levels of EI are more likely to show a greater capacity for learning, deal with pressure more effectively, communicaz te in interesting and assertive ways and recognise the strengths and weaknesses of others. Overall, the trend is that they are more productive and satisfied in the workplace. A further point is that the EI of students, employees and managers can be enhanced with training.

When recruiting to an IPC post, the interview is place where a candidate’s EI can be assessed. Emotional competence is thought to be important for successful social interactions (Lopes et al., 2004). In essence, an interview is a social interaction between two or more individuals who want to exchange information or signals about their qualities. Interviews are often seen as ‘high stakes’ experiences that can invoke any number of emotions, that include excitement, panic, doubt, desperation, frustration, stress, embarrassment, impatience, relief, agitation, nervousness, pride, joy. When a candidate is aware of these emotions, this can provide the motivation to regulate and manage them appropriately and proportionately. In relation to the other two components of EI, an interviewer can witness a candidate’s empathy, social and people skills by the way they listen and communicate in the interview. Content, what they say, is of course important, but how this is framed, fluent speech, a confident and modulating voice, direct eye contact, an expressive face with smiles and nods, are associated with EI and successful candidates (Muralidhar et al., 2016). An example of questions that can help gain an insight into a candidate’s EI is

Describe an example when you have had to be confrontational to achieve results. What did you do and how was it received?

What is one of your proudest achievements?

In the second example, not only does the answer reveal a lot about how they see success. But it is interesting to note whether they select a solo or team achievement.

Once in post, any rudimentary online search will identify a plethora of tools, from authentic sources, that attempt to measure an individual’s EI. Some incur a cost, but many are free. Some are text based, others are based on a series of images. Two examples come from Psychology Today, 2022 and Psych Tests, 2022 and these appear in the reference list. While these may seem a little over-simplified, they are a convenient way to introduce EI to an IPC team and start a discussion about where their own EI stands. That is, begin a discussion on the value of self-awareness, empathy and how well they manage their emotions when placed in stressful situations. As part of revalidation the NMC promotes a culture of reflection by asking registrants to record a minimum of five written reflections on their continuing professional development (CPD) activity and/or feedback and/or a practice related event (NMC, 2021). Reflection allows nurses to promote a flexible approach to care; equips them with problem-solving skills through systematic reasoning and makes them more inclined to monitor and enhance their professional competence (Schumann Scheel et al., 2017). Reflection can be an individual pursuit, but the NMC advise that reflecting in groups, teams and multi-professional settings is an excellent approach to advance collective wisdom. It can also be an accomplished way to build EI. The team can bring their experiences of conflict to a debrief. Using the elements of EI as framework (Table 2), a reflective discussion can be had on the circumstances, the emotions at the time, how well these were regulated how the antagonist may have felt, along with actions and outcomes.

## Conclusion

Tough and increasingly punitive attitudes, have introduced a new component into the work of IPC teams. But if as Almost (2016) suggests positive, collaborative, working relationships are a necessary feature of enhanced patient outcomes, productive workforces and safe cultures, individuals in the IPC Team need support and training for how they manage conflict. The IPC competency framework is a multicomponent tool that acknowledges the many facets of the ICP role. However, I would argue that the default is still to see IPC as a hard, deductive science. More can be done to see IPC as a social science that is grounded in human behaviour. Vis-à-vis compliance with best practice seems to be more problematic than understanding what best practice looks like. Nurses who lead with EI demonstrate a sensitivity to their own and other people’s psychological health and well-being, and are more able to direct others towards common goals. Whether or not EI is stated, explicitly, in the framework introducing the concept to IPC where hitherto it has been absent, seeking it as an attribute when recruiting staff, then enhancing it through training and reflection, would be a good thing.
